# 

**Published:** 1915-06-26

**Authors:** 


### CONTENTS.

| | PAGE |
|---|---|
| **H.R.H. the Prince of Wales** ... ... ... | 261 |
| **The Responsibility of Names** ... ... ... | 262 |
| **Hospital and Institutional News** ... ... ... | 263 |
| The New Secretary of Hampstead General Hospital—The Medical Service of the German Army—The St. George's Hospital Site—Soldiers' Diet in the Coventry Hospital—Leicester Workmen and the Wounded—Postponing Extensions till after the War—The Out-Patient Problem at Wolverhampton—Nerve Cases in the Army—Regiments and the County Hospitals—The New Wing at Haslar—Hospitals and the Coal Supply—The Exchange of Medical Officers and Disabled Prisoners—Transfers to the Royal Army Medical Corps—Something Worth Seeing—The Mental Hospital Roll of Honour—Indian Ruler's Gift of a Hospital for Officers—Women and the Health of the Civil Population—A Model of Paper and Printing—A Reconstituted Sanatorium—Medical Inspection and Anæmic School Children—The Badge Question—Increase of Medical Staff at a Poor-Law Infirmary—Another Commission for a Hospital Officer—The late Captain Garnier and East Sussex Hospital—The British Hospital in Moscow—A Reduction in a Drug Account—This Week's Drug Market. | |
| **Hospitals and the Spirit Tax** ... ... ... | 269 |
| The Government Amendment to the Finance Bill. | |
| **With the South African Medical Corps** ... | 270 |
| Medical Organisation in German S.W. Africa: The Transport Problem. | |
| **Anti-Tuberculosis Organisation in London** ... | 271 |
| Comprehensive Scheme for Adults and Children. | |
| **Voluntary Field Hospitals and Military Hygiene** ... ... ... ... ... ... | 272 |
| **A Medical Causerie** ... ... ... ... ... | 273 |

[*See over.*]

### CONTENTS—(*continued*).

PAGE

**Round the World** ... ... ... ... ... 274

Fellow-Workers and the Roll of Honour.

**Hospital Investments and Trustees** ... ... 275

A Practical View of Depreciation.

**Hospital Architecture and Construction** ... 277

Observation Wards in Isolation Hospitals: The Cruciform Plan.

**Buildings Contemplated and in Progress** ... 279

**The London Panel Committee** ... ... ... 279

Hospitals and Local Panel Associations.

**Mentioned in Dispatches** ... ... ... ... 280

Army Service Corps (Attd. R.A.M.C.).

Medical Service and R.A.M.C.

Royal Army Medical Corps (T.F.).

British Red Cross Society.

Civil Hospitals Reserve.

Canadian Army Medical Corps.

PAGE

**Medical Appointments** ... ... ... ... ... vi

**Doctors' Wills** ... ... ... ... ... ... ... vi

**The Institutional Worker** ... ... (Suppl.) 1

National Insurance Work: Answer to May Question.

Practical Matters: Annual Cleaning.

Question for June.

Questions Invited.

Enquiries and Answers.

The Sick and In Need: Required for Invalid.

Employment and Training: Health Appointments—Nursing Abroad—London Institutes for the Supply of Midwives—Three Months' Training.

Miscellaneous: Hospitals and Aircraft Bombs—The Duties of a Ward Sister and Staff Nurse—Collecting-Box Makers.

Acknowledgments.

